# Sensory deficiencies correlate with tau protein and dementia

**DOI:** 10.3389/fnins.2026.1754329

**Published:** 2026-03-03

**Authors:** Marina Avila-Villanueva, Félix Hernández, Jesús Avila, Germán Plascencia-Villa, George Perry

**Affiliations:** 1 Departamento de Psicología Social, del Trabajo y Diferencial, Facultad de Psicología, Universidad Complutense de Madrid, Madrid, Spain; 2 Centro de Biología Molecular Severo Ochoa (CSIC-UAM), Madrid, Spain; 3 Center for Networked Biomedical Research on Neurodegenerative Diseases (CIBERNED), Madrid, Spain; 4 Department of Neuroscience, Developmental and Regenerative Biology, The University of Texas at San Antonio, San Antonio, TX, United States

**Keywords:** cognitive dyfunctions, dementia, sensory, sensory dysfunction, tau

## Abstract

Sensory decline is a common feature of aging and an early sign of a high risk of developing neurodegenerative diseases. Abnormal protein deposits of tau are also observed in sensorial areas in early stages of Alzheimer’s disease and related dementia (ADRD), indicating that these two features are associated with common neuropathological changes in the affected brain areas. Alterations in taste and smell are evident in subjects with cognitive decline, but sensory decline is perceived in olfaction, vision, hearing (at early times of degeneration), and even touch, which correlates with disease progression. Consequently, affected individuals may suffer from varying altered behaviors that emerge from the declined capability to process and perceive information, suggesting that differences in sensory perception of the environment may play a key role in explaining these behavioral variations in subjects with cognitive impairment. This commentary discusses some of the alterations in sensory functionality and how these could contribute to the development of neurodegenerative disorders, such as ADRD.

## Introduction

Age-related sensory decline across the five senses has been well-documented (Cavazzana et al., 2018). This decline is influenced by changes in the nervous system, which is responsible for regulating sensory function. Brain aging is considered the primary risk factor for the development of neurodegenerative disorders such as Alzheimer’s disease (AD; Avila, 2024). Among the molecular contributors to AD, tau protein plays a significant role (Avila et al., 2004). As discussed below, sensory impairments have been observed in individuals with AD and, in some cases, these deficits may emerge during the preclinical stage of the disease. This brief report explores the hypothesis that sensory dysfunction could serve not only as an early symptom but also as a potential risk factor for the development of AD. In particular, we examine the possible involvement of tau protein in age-related sensory impairments. Current knowledge about the role of tau in sensory function is limited, especially based on studies in tau knockout (k.o.) mice (Harada et al., 1994; Dawson et al., 2001; de Gomez Barreda et al., 2010; Beauchamp et al., 2018). Also, there is some evidence suggesting that tau influences neural plasticity (Rodriguez et al., 2020) and may play a role in sensory decline, as shown in various mouse models of tauopathies (Macknin et al., 2004; Mellone et al., 2013; Park et al., 2018; Wang and Wu, 2021; Meftah et al., 2024). In this context, we aim to discuss the potential contribution of tau protein to aging-related sensory deficits, and how these deficits may be implicated in the early stages of AD pathogenesis.

## Individual differences in humans related to their interaction with the environment

The concept of “precision medicine” is gaining increasing attention, aiming to identify individual characteristics that may influence a person’s response to external stimuli or specific medical treatments (Hampel et al., 2019). Humans do not interact with their environment in a uniform way, as individual characteristics contribute to variations in how we perceive our surroundings. Specific personal traits influence these differences in environmental perception. We experience the world through our senses, and the information received—unique to each individual—can be processed, stored, and used to shape future behavior. In fact, our personal perception of the environment can help develop a memory system that influences planned behavior in both the present and future (Budson et al., 2022). This behavior can be reflexive or emotionally driven (Orpwood, 2017). Sensory information is crucial in shaping individual responses since we relate to our surroundings through our senses. As noted by F. Galton long time ago, differences in sensory function can significantly impact behavior (Galton, 1883).

## Qualia

To account for individual differences in sensory information, the concept of “Qualia” emerged in the late-20th century (Jackson, 1982; Ramachandran and Hirstein, 1997; Tye, 2006; Browning, 2024). The American Psychological Association (APA) defines Qualia as the qualities that shape the nature of mental experiences, such as sensations or perceptions, distinguishing them from other experiences. Examples of qualia include the experience of pain, the taste of wine, the color of the sky, the smell of a flower, or the sound of a musical instrument, indicating that qualia may modulate the information received by the senses (Skokowski, 2022), in different ways. At the cellular level, various types of neurons are involved in different sensory pathways, and variations in specific neuronal proteins, such as tau protein, may play a role in these pathways.

Several questions regarding qualia have arisen across various fields, such as philosophy, psychology, and neuroscience. These questions include: (a) Do qualia exist? (b) If qualia exist, can they be measured? (c) Do animals, such as mammals, experience qualia? (d) Is there a pathology related to qualia? Regarding point (a), different disciplines have offered various definitions of qualia, and some have raised objections. One notable objection is the ‘absent qualia’ hypothesis (see, for instance, Tye, 2006). Additionally, qualia are often described as individual and subjective, which complicates their measurement (Jackson, 1982). However, neuroscience has explored subjective experiences, such as subjective cognitive decline (SCD) in humans, which can be measured across different levels (Avila-Villanueva et al., 2018; Jessen et al., 2020; Jones and Hunt, 2023). For point (b), we focus on the concept of ‘sensing qualia’ (Skokowski, 2022), proposing that sensory perceptions like taste, vision, hearing, or smell could yield measurable qualitative differences through analysis of sensory responses. While the definition of ‘sensing qualia’ may not be universally accepted, it will serve as the basis for our discussion. As for point (c), we propose that qualia, or related characteristics, might exist in mammals and could be studied using animal models. Mammals exhibit measurable sensory responses to external stimuli, suggesting they might experience qualia or similar phenomena.

Three recent papers explore various aspects of qualia. One discusses electromagnetic field theories of qualia, suggesting that different brains might generate distinct colors, tastes, or sounds, possibly explained by a ‘spike code’ (Jones and Hunt, 2023). Another paper proposes that electromagnetic fields generated by neurons influence individual sensory perceptions (Bond, 2022). A third paper introduces mathematical methods to design new experiments for measuring colors, tastes, and sounds (Tsuchiya et al., 2022). However, neuronal interactions should also be examined at the cellular and molecular levels using molecular biology techniques. Although qualia are often defined as the subjective qualitative aspects of an experience, these subjective elements can also derive from individual characteristics., such as genetic factors shared among members of a population. In this context, Moriguchi et al. (2025) recently analyzed color similarity judgments in large cohorts of children and adults. Their findings showed that color qualia structures emerge early in childhood, yet age-related differences appear in how some colors are perceived in adulthood. Future research could explore whether these age-related perceptual differences correlate with structural or molecular changes occurring during the aging process.

Differences between individuals’ brains may stem from genetic variability. For example, behavioral differences in mice responding to the same stimuli, such as odors, are often observed. These variations in olfactory sensitivity may lead to different perceptions of reality and distinct behaviors. To address this, researchers use the same mouse strains in experiments, seeking a homogeneous genetic background to account for variations in olfactory receptors (Gilbert et al., 1986; Wang et al., 1993; Bansal et al., 2021). In humans, genetic variations in olfactory perception have been identified. Studies on more genetically homogeneous populations, such as Icelandic groups, have been conducted to analyze behaviors associated with olfactory perception for behavioral testing (Helgason et al., 2005). Finally, concerning point (d) on potential qualia-related pathologies, defects in sensory pathways could lead to qualia dysfunction. For instance, Kandinsky’s synesthesia, where sounds and colors are perceived interchangeably, is an example of such a dysfunction (Just, 2017). More recently, the existence of a functional bridge between vision and touch has been proposed (Hedger et al., 2026).

## Role of neural proteins in sensory perception

At the cellular level, it is important to consider the function of various neurons involved in distinct sensory pathways, with different proteins or isoforms of a single protein contributing to the processing of external stimuli. Additionally, individual variations in sensory reception may occur, influenced by neuronal proteins involved in sensory processing. One example is tau protein, which has been implicated in sensory reception and processing (Lee et al., 2001). For instance, the absence of tau protein in tau knockout mice leads to olfactory deficits (Beauchamp et al., 2018).

## Presence of different tau isoforms in sensory neurons

Distinct types of sensory neurons involved in different senses express specific tau isoforms, suggesting specialized molecular adaptations in sensory processing. In vision, the presence of big tau isoforms containing exon 4a has been reported in the retina (Chiasseu et al., 2016; Fischer et al., 2024; Shi et al., 2024). Additionally, rare post-translational modifications of tau, such as citrullination, have been detected, particularly under pathological conditions (Nicholas, 2013).

**Figure 1:**
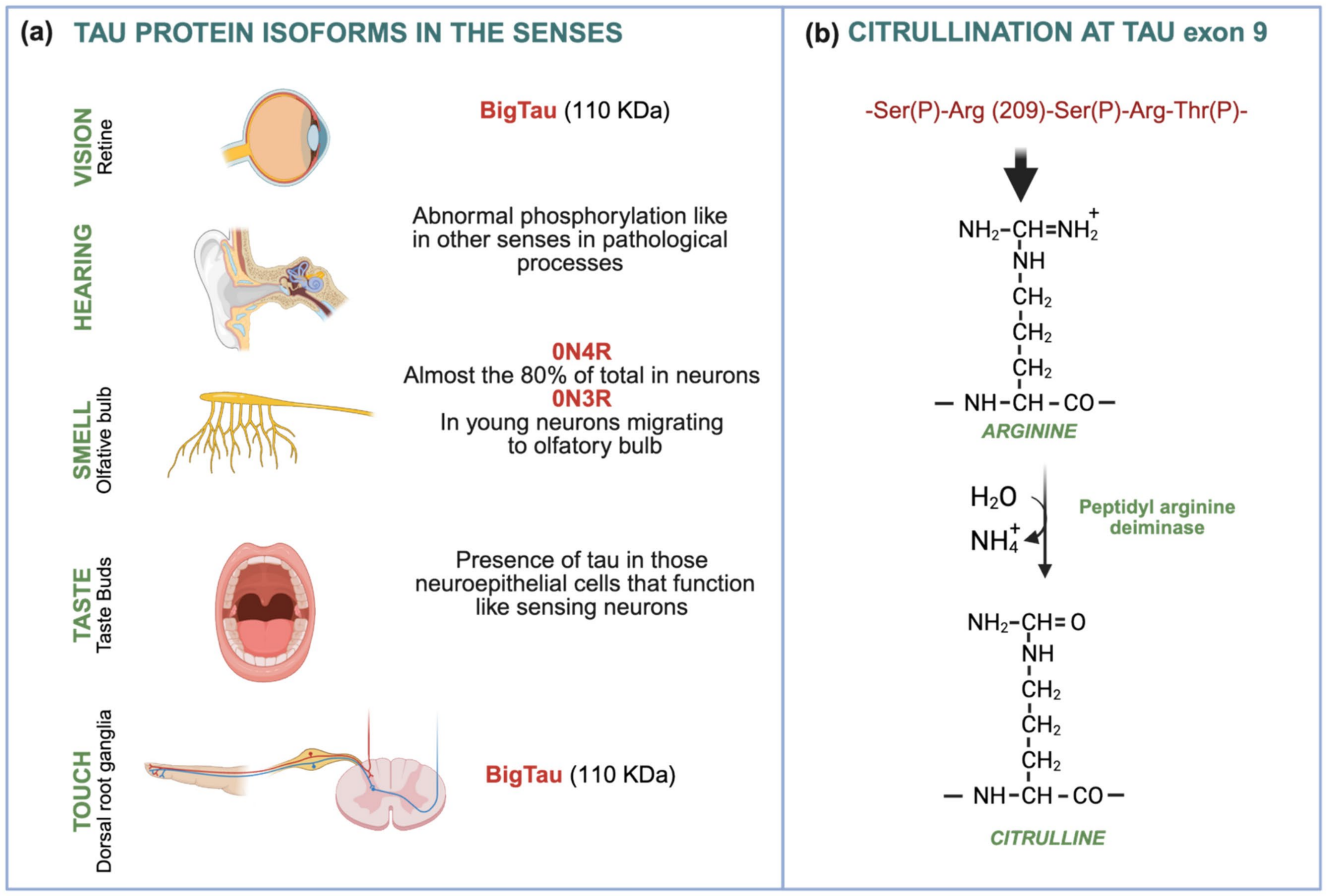
Presence of different tau isoforms across sensory systems. **(a)** In vision and touch, the big tau isoform—predominantly expressed in the peripheral nervous system (PNS)—can be detected. In the olfactory system, there is a reduction in tau isoforms containing exon 2 or exons 2 and 3. In taste, tau is found in neuroepithelial cells within taste buds. In hearing, as in other senses, tau phosphorylation by various kinases is observed under pathological conditions. **(b)** In the retina, the tau sequence encompassing exon 9 (residues 208–212) can undergo post-translational modification either by kinases (phosphorylation) or by peptidyl arginine deiminases (citrullination). The mechanism of citrullination at arginine 209 is illustrated. References are provided in the text.

In touch, big tau isoforms (approximately 100 kDa) are expressed in sensory neurons responsible for transmitting stimuli such as touch, pain, and temperature (Oblinger et al., 1991). Dorsal Root Ganglion (DRG) neurons display a distinctive morphology with two branches connecting the periphery to the spinal cord, serving as a key interface between the Peripheral Nervous System (PNS) and the Central Nervous System (CNS; Donovan et al., 2025). In smell, within the olfactory bulb, the ON4R tau isoform accounts for roughly 80% of total tau isoforms, while the ON3R tau isoform predominates in young olfactory neurons (Tuerde et al., 2018). These findings suggest that aging or neuronal maturation may be accompanied by a decrease in tau isoforms containing exon 2 or exons 2 and 3. Smell and taste are closely related senses (Spence, 2015). In mouse models, tau protein is expressed in the neuroepithelial cells of taste buds, which function similarly to sensory neurons (Kim et al., 2024a). Tau is mainly found in type II and III taste cells, whereas type I cells are enriched in APP (amyloid precursor protein; Kim et al., 2024a). In hearing, as in other sensory modalities, differences in tau phosphorylation have been described, some of which parallel those observed in neurodegenerative disorders (Xu et al., 2019; Wang et al., 2022). Taken together, these findings indicate that the differential expression and modification of tau isoforms across sensory systems may contribute to both the functional specialization of sensory neurons and their selective vulnerability in tauopathies. Key examples of these isoform-specific distributions are summarized in Figure 1a.

## Correlations of senses dysfunctions with neurodegenerative disorders

Alzheimer’s disease (AD), the most common form of dementia, is mainly characterized by two histopathological abnormal protein structures: senile plaques, made up of amyloid-beta (Aβ) aggregates observed in the interstitial spaces of neuronal cells, and neurofibrillary tangles, consisting of hyperphosphorylated tau protein inside of neurons. These structures are commonly used as histopathological markers for diagnosis and monitoring the progression of the disease. Deposition of Aβ typically precedes neurofibrillary and neuritic changes, with initial changes in the frontal and temporal lobes. Whereas, neurofibrillary tangles and neuritic degeneration begin in the medial temporal lobes and hippocampus and progressively spread to the neocortex during the course of the disease (Masters et al., 2015).

After the pioneer work of Braak and Braak (Braak and Braak, 1991), it became clear that tau pathology correlates with critical higher-order cognitive processes such as attention, learning, and memory, even at early disease Braak stages. Sensory dysfunctions could contribute to these correlations in neurodegenaration. In the case of tau pathology, it mainly arises from a toxic gain of function of modified tau protein rather than from its absence. However, studies using tau knockout mouse models have highlighted the importance of tau protein localized in dendritic spines for processes involved in the interaction of neural cells—including sensory neurons—with the external environment (Pallas-Bazarra et al., 2016). Additionally, recent research has suggested that sensory experiences such as music (hearing) may play a role in the development of emotional memory (Moltrasio and Rubinstein, 2025).

## Sensory deficits in Alzheimer disease

Memory problems are typically one of the early signs of AD, as well as decline in non-memory aspects of cognition. However, a decline or alterations of the senses: sight, hearing, smell, taste, and touch, is another early sign of AD and related dementia that are frequently overlooked but that could indicate probable initiation of neurodegeneration. Tau protein is a neuronal protein involved in several functions and dysfunctions (Iqbal et al., 2016), and it may contribute to a potential ‘spike code’ (Jones and Hunt, 2023) due to its presence in dendritic spines (Ittner et al., 2010; Pallas-Bazarra et al., 2016), where it could regulate synaptic transmission. Therefore, tau pathology is not only linked to dementia or impaired consciousness (Avila et al., 2004) but also to sensory dysfunctions, including smell, vision, hearing, touch, and taste.

## Tau pathology in different senses

### Olfactory impairment

Olfactory impairment has been observed in AD patients (Attems and Jellinger, 2006; Klein et al., 2021), and even during the earlier stage of mild cognitive impairment (MCI) (Risacher et al., 2017). This impairment worsens along the AD continuum (Attems et al., 2005), as demonstrated by smell tests (Bond and Notides, 1988; Growdon et al., 2015). Therefore, assessing the sense of smell may serve as an early marker for dementia development (Tian et al., 2022). Recent research has demonstrated how odor information is processed in the brain by recording the activity of a large number of individual neurons. In addition to olfactory regions like the piriform cortex, other brain areas not typically associated with smell—such as the amygdala, entorhinal cortex, hippocampus, and parahippocampal cortex—may play a role in odor identity, odor valence (how pleasant the odor is), and odor recognition (Kehl et al., 2024). Given that tau pathology in AD is linked to the entorhinal cortex and hippocampus, a connection between olfactory function and AD can be suggested, as previously proposed. Tau pathology can be observed in the olfactory bulb, with odor impairment worsening as AD progresses (Attems et al., 2005; Diez et al., 2024), as measured through smell tests (Bond and Notides, 1988; Growdon et al., 2015). Tau pathology is associated with olfactory dysfunction (Klein et al., 2021), which, as mentioned earlier, is observed not only in AD but also in MCI. This tau pathology is linked to the phosphorylation of tau protein at residue 181, as suggested by Klein et al. (2021).

### Vision impairment

Vision impairment has been proposed as a potential marker for the development of dementia (Ehrlich et al., 2022) and has recently been recognized as one of the 14 modifiable risk (Livingston et al., 2024) factors for dementia. Studies have shown a higher incidence of dementia in older adults with poor visual capacity (Davies-Kershaw et al., 2018; Lee et al., 2020). While the occipital lobe, home to the visual cortex, typically remains undamaged in AD, the link between vision impairment and dementia has focused on other areas, such as the retina, where pathological changes have been observed in AD (Koronyo et al., 2023). In fact, retinal pathology may play a more significant role in AD than cortical changes (Hart et al., 2016). This retinal pathology has also been noted in mild cognitive impairment (MCI), making it a potential early marker for dementia. Additionally, presenile dementia has been linked to a form of cortical blindness known as Heidenhain’s syndrome (Meyer et al., 1954). Tau pathology is associated with visual dysfunction, affecting both the retina and the optic nerve (Ho et al., 2012; Walkiewicz et al., 2024), and is correlated with tau phosphorylation at residue 217. This phosphorylation occurs very early in the AD continuum (Avila and Perry, 2021). As previously noted, a post-translational modification of the tau protein—citrullination at arginine residue 209—is increased in tau pathology associated with visual dysfunction, which correlates with the development of AD (Nicholas, 2013; Figure 1b).

### Tactile and gustatory impairment

Studies have shown a significant reduction in total taste scores, as well as for individual tastes on either side of the tongue, in AD patients compared to non-demented controls. Aging is associated with a decline in taste sensitivity, and this effect is also observed in a fly model expressing beta-amyloid peptides (Brown et al., 2024). Similar to smell and vision, differences in taste perception have been noted between patients with MCI and healthy controls (Steinbach et al., 2010). Therefore, changes in taste, like those in smell and vision, are proposed to be linked to dementia progression. Dementia also affects the sense of touch, with a 1.6% higher risk of dementia associated with impaired tactile sensation (Brenowitz et al., 2022). Additionally, altered processing of pain and temperature has been observed in dementia (Fletcher et al., 2015). As indicated, touch involves sensory neurons connected to both the Central Nervous System (CNS) and the Peripheral Nervous System (PNS). An example of such neurons are Dorsal Root Ganglia (DRG) neurons, which relay sensory stimuli from the PNS to CNS neurons. DRG neurons may express small tau isoforms at certain stages; however, a defining feature of these cells is the later emergence of the big tau isoform (Nothias et al., 1995). Small tau isoforms, missing exon 4a, are present in CNS, whereas big tau, containing exon 4a, is present in PNS (Fischer, 2022). In the CNS, small tau isoforms are prevalent, while the big tau isoform is found mainly in the PNS (Fischer and Baas, 2020). The big tau isoform may play a protective role in tau-related pathology (Fischer and Baas, 2020), suggesting that touch might be less connected to dementia or tau pathology. On the other hand, tau pathology in the spinal cord has been observed in Alzheimer’s disease (Schmidt et al., 2001; Lorenzi et al., 2020), specifically involving small tau isoforms. Therefore, the presence of distinct tau isoforms in different sensory pathways could contribute to varied roles in dementia. Nonetheless, touch remains an important means of communication with older adults with dementia, as it is less affected by sensory decline (Gleeson and Timmins, 2004). In a tauopathy mouse model (Pennanen et al., 2004), it was demonstrated that expressing mutated human tau (Tau P301S) leads to accelerated extinction of conditioned taste aversion. Interestingly, phosphorylated tau has been observed in taste bud cells (types II and III) in AD mouse models (Kim et al., 2024a), whereas amyloid precursor protein (APP) is mainly present in taste bud cell type I. Moreover, other AD mouse models, such as APP/PS1 mutant transgenic mice, have shown selective dysfunction in peripheral taste perception (Wood et al., 2020).

### Auditory dysfunction

Hearing is likely the most significant sense linked to dementi and its prodromal stages (Swords et al., 2018). According to the World Health Organization, individuals with hearing loss are twice as likely to develop dementia compared to those without it, and in cases of severe hearing loss, the risk increases nearly fivefold (Hardy et al., 2016). Hearing loss is considered a major risk factor for dementia (Thomson et al., 2017). Age-related hearing impairment, or presbycusis, is the most common hearing disorder in older adults and is a modifiable risk factor for both dementia and AD (Panza et al., 2015). Progressive hearing decline has been associated with increased *β*-amyloid and tau deposition in individuals with age-related hearing loss (Zheng et al., 2022). Additionally, a connection between tau pathology and hearing impairment has been reported (Park et al., 2018; Wang et al., 2022). Neurons in the orbital frontal cortex (OFC) are involved in auditory responses, which are shaped by both lemniscal and non-lemniscal pathways. The lemniscal pathway directs auditory information to the auditory cortex, while the non-lemniscal pathway connects to other brain regions, including the hippocampus (Nadhimi and Llano, 2021; Paciello et al., 2021). The hippocampus, a key site of tau pathology in AD, experiences increased tau phosphorylation at serine 396, which promotes long-term depression (LTD; Kimura et al., 2014; Regan et al., 2015). Hippocampal LTD has been implicated in synaptic dysfunction in AD (O'Riordan et al., 2018). Additionally, the induction of LTD increases tau phosphorylation at residues 181 and 217 (Zhang et al., 2022). These examples highlight how tau protein-related dysfunction can affect sensory processing. Tau pathology, driven by changes such as phosphorylation or aberrant aggregation (Buee et al., 2000; Avila et al., 2004; Ittner et al., 2010), may contribute to sensory deficits. Other proteins involved in sensory receptors or external stimulus transmission might also play a role in the variability of “sensing qualia.” Recently, additional studies linking hearing loss and dementia have been published (Kolo et al., 2025). Moreover, hearing loss has been proposed as an early neurodegenerative biomarker for disorders such as Alzheimer’s disease, assessed through noise-based tests, alongside other well-established biomarkers, including plasma levels of NfL, GFAP, and tau p217 (Bekena et al., 2025).

## Discussion

**Figure 2:**
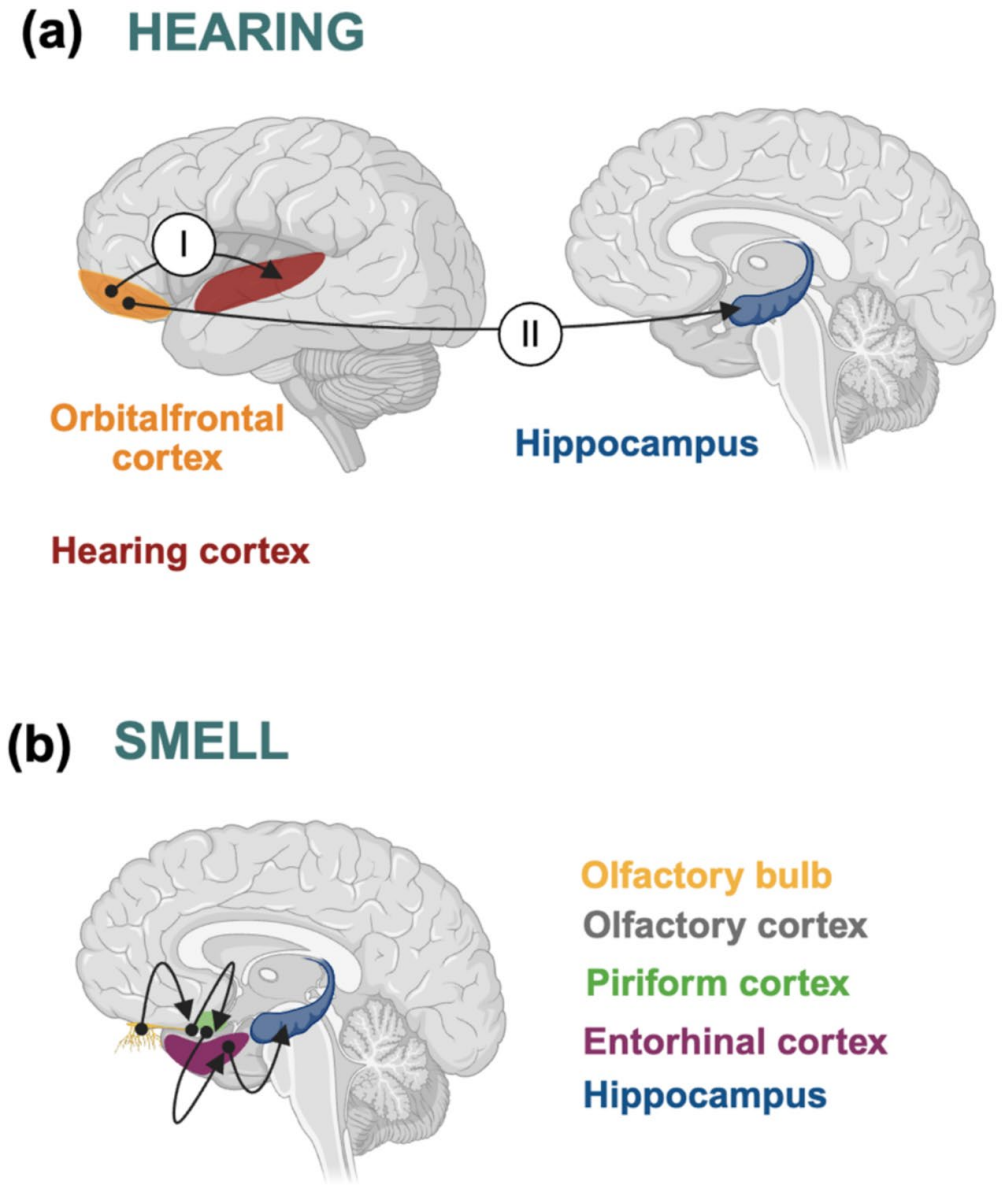
Sensory deficits, tau pathology, and the development of Alzheimer’s disease, the most prevalent form of dementia. **(a)** Neurons in the orbitofrontal cortex (orange) are involved in auditory processing via two pathways: one (I) connecting with the auditory cortex (red) and another (II) linking to the hippocampus (blue). Disruptions in the second pathway may correlate with Tau pathology in the hippocampus, a hallmark of Alzheimer’s disease. **(b)** Olfactory information from the olfactory bulb (yellow) is transmitted to the olfactory cortex (gray), piriform cortex (green), entorhinal cortex (purple), and hippocampus (blue). As noted in **(a)**, Tau pathology in the hippocampus is a defining characteristic of Alzheimer’s disease.

Environmental perception is mediated through the senses, which, at the cellular level, involve various types of neurons expressing distinct proteins or specific isoforms of the same protein. These proteins (or isoforms) contribute to sensory pathways and may be implicated in neurodegenerative disorders like dementia. Differences in how individuals perceive their environment may be linked to the expression of particular protein isoforms, such as those of tau. For example, impairments in the olfactory or hearing senses (see Figure 2) have been associated with tau pathology in neurons that express small tau isoforms. In contrast, the presence of big tau isoforms in some touch-related neurons may offer partial protection against certain disorders. Additionally, the ability to recognize environmental stimuli is connected to cognitive processes like attention to important features, learning, and memory (Budson et al., 2022). These characteristics differ among individuals due to their connection with sensory pathways. For instance, genetic variations, epigenetic modifications, or distinct haplotypes (such as those for the MAPT gene, which encodes tau protein; Caffrey and Wade-Martins, 2007), can affect the levels or types of tau isoforms expressed in different brain areas. Indeed, different tau isoforms are expressed in the Central Nervous System (CNS) and the Peripheral Nervous System (PNS), which may contribute to the higher vulnerability of CNS regions, such as the cortex and hippocampus, to tau pathology compared with the PNS. These differences are not due to modifications of residues such as tyrosine 29, which may play a role in the development of tau pathology (Kim et al., 2024b), but rather to the presence of specific exons, such as exon 4a, which is predominantly expressed in the PNS and constitutes a major component of the isoform known as “big tau” (Fischer and Baas, 2020; Fischer, 2022). Indeed, it has been suggested that “big tau” may be unable to aggregate in the same manner as smaller tau isoforms (Avila, 2000). Moreover, a very recent study shows that the full-length tau isoform containing all 16 exons has a very low assembly capacity (Valles-Saiz et al., 2025). Identifying these genetic or epigenetic variants could enhance the clinical diagnosis of neurodegenerative disorders, especially if specific tau isoforms are associated with dysfunction in particular sensory neurons. In the context of neurodegenerative diseases like Alzheimer’s disease, which progresses along a continuum (Avila-Villanueva et al., 2020; Avila and Perry, 2021), it would be valuable to monitor the progression of sensory impairments associated with various aspects of tau pathology. Combining sensory function assessments (such as smell or hearing tests, see Figure 2) with cognitive tests for memory and attention could support early diagnosis of dementia.

## Conclusion

Human perception of the environment is a complex, multifactorial process involving the detection of external information through the senses (qualia). This process is influenced by sensory input as well as the overall brain state (e.g., wakefulness, not discussed here, see Zaretskaya (2024)). Deficits in this process may increase the risk of developing dementia. We have highlighted the potential role of sensory deficits in dementias such as Alzheimer’s disease, as well as the involvement of Tau protein, a well-established marker in various tauopathies, in neurodegeneration. Currently, the most significant sensory deficit risk for Alzheimer’s disease is hearing impairment, though recent findings also suggest a possible role for olfactory impairment (see Figure 2). The good news is that these risks are modifiable and could potentially be reversed to delay the development of the disease, provided that proper clinical studies, precision medicine, precision psychology, and sensory tests are properly conducted.

## References

[ref1] Attems J. Jellinger K. A. (2006). Olfactory tau pathology in Alzheimer disease and mild cognitive impairment. Clin. Neuropathol. 25, 265–271. 17140156

[ref2] Attems J. Lintner F. Jellinger K. A. (2005). Olfactory involvement in aging and Alzheimer's disease: an autopsy study. J. Alzheimer's Dis 7, 149–157. doi: 10.3233/jad-2005-7208, 15851853

[ref3] Avila J. (2000). Tau aggregation into fibrillar polymers: taupathies. FEBS Lett. 476, 89–92. doi: 10.1016/s0014-5793(00)01676-8, 10878257

[ref4] Avila J. (2024). Delaying brain aging or decreasing tau levels as strategies to prevent Alzheimer's disease: in memoriam of mark a. smith. J. Alzheimer's Dis 100, S265–S270. doi: 10.3233/JAD-240500, 39058443

[ref5] Avila J. Lucas J. J. Perez M. Hernandez F. (2004). Role of tau protein in both physiological and pathological conditions. Physiol. Rev. 84, 361–384. doi: 10.1152/physrev.00024.2003, 15044677

[ref6] Avila J. Perry G. (2021). A multilevel view of the development of Alzheimer's disease. Neuroscience 457, 283–293. doi: 10.1016/j.neuroscience.2020.11.015, 33246061

[ref7] Avila-Villanueva M. Gomez-Ramirez J. Maestu F. Venero C. Avila J. Fernandez-Blazquez M. A. (2020). The role of chronic stress as a trigger for the Alzheimer disease continuum. Front. Aging Neurosci. 12:561504. doi: 10.3389/fnagi.2020.561504, 33192456 PMC7642953

[ref8] Avila-Villanueva M. Maestu F. Fernandez-Blazquez M. A. (2018). Internal consistency over time of subjective cognitive decline: drawing preclinical Alzheimer's disease trajectories. J. Alzheimer's Dis 66, 173–183. doi: 10.3233/JAD-180307, 30248053

[ref9] Bansal R. Nagel M. Stopkova R. Sofer Y. Kimchi T. Stopka P. . (2021). Do all mice smell the same? Chemosensory cues from inbred and wild mouse strains elicit stereotypic sensory representations in the accessory olfactory bulb. BMC Biol. 19:133. doi: 10.1186/s12915-021-01064-7, 34182994 PMC8240315

[ref10] Beauchamp L. C. Chan J. Hung L. W. Padman B. S. Vella L. J. Liu X. M. . (2018). Ablation of tau causes an olfactory deficit in a murine model of Parkinson's disease. Acta Neuropathol. Commun. 6:57. doi: 10.1186/s40478-018-0560-y, 29976255 PMC6032546

[ref11] Bekena S. Singh R. K. Zhu Y. Cruchaga C. Arnold S. E. Ances B. M. . (2025). Hearing loss, plasma neurodegenerative biomarkers, and cognitive function: independent and additive effects. J. Alzheimer's Dis 108, 661–670. doi: 10.1177/13872877251378675, 40961167 PMC12790462

[ref12] Bond E. (2022). The contribution of coherence field theory to a model of consciousness: electric currents, EM fields, and EM radiation in the brain. Front. Hum. Neurosci. 16:1020105. doi: 10.3389/fnhum.2022.1020105, 36760225 PMC9903675

[ref13] Bond J. P. Notides A. C. (1988). A chemical kinetic model for ligand binding to identical and independent binding sites in vivo. Anal. Biochem. 175, 238–251. 2854373 10.1016/0003-2697(88)90384-3

[ref14] Braak H. Braak E. (1991). Neuropathological stageing of Alzheimer-related changes. Acta Neuropathol. 82, 239–259. 1759558 10.1007/BF00308809

[ref15] Brenowitz W. Robbins N. Strotmeyer E. Yaffe K. (2022). Touch sensations is an understudied predictor of dementia risk in older adults. Innov. Aging 6, 151–151. doi: 10.1093/geroni/igac059.603

[ref16] Brown E. B. Lloyd E. Riley R. Panahidizjikan Z. Martin-Pena A. McFarlane S. . (2024). Aging is associated with a modality-specific decline in taste. iScience 27:110919. doi: 10.1016/j.isci.2024.110919, 39381735 PMC11460507

[ref17] Browning J. (2024). The history of qualia and C.I. Lewis’ role in it. Br. J. Hist. Philos. 32, 173–193. doi: 10.1080/09608788.2023.2233094

[ref18] Budson A. E. Richman K. A. Kensinger E. A. (2022). Consciousness as a memory system. Cogn. Behav. Neurol. 35, 263–297. doi: 10.1097/WNN.0000000000000319, 36178498 PMC9708083

[ref19] Buee L. Bussiere T. Buee-Scherrer V. Delacourte A. Hof P. R. (2000). Tau protein isoforms, phosphorylation and role in neurodegenerative disorders. Brain Res. Brain Res. Rev. 33, 95–130. doi: 10.1016/s0165-0173(00)00019-9 10967355

[ref20] Caffrey T. M. Wade-Martins R. (2007). Functional MAPT haplotypes: bridging the gap between genotype and neuropathology. Neurobiol. Dis. 27, 1–10. doi: 10.1016/j.nbd.2007.04.006, 17555970 PMC2801069

[ref21] Cavazzana A. Rohrborn A. Garthus-Niegel S. Larsson M. Hummel T. Croy I. (2018). Sensory-specific impairment among older people. An investigation using both sensory thresholds and subjective measures across the five senses. PLoS One 13:e0202969. doi: 10.1371/journal.pone.0202969, 30148857 PMC6110574

[ref22] Chiasseu M. Cueva Vargas J. L. Destroismaisons L. Vande Velde C. Leclerc N. Di Polo A. (2016). Tau accumulation, altered phosphorylation, and Missorting promote neurodegeneration in Glaucoma. J. Neurosci. 36, 5785–5798. doi: 10.1523/JNEUROSCI.3986-15.2016, 27225768 PMC6601840

[ref23] Davies-Kershaw H. R. Hackett R. A. Cadar D. Herbert A. Orrell M. Steptoe A. (2018). Vision impairment and risk of dementia: findings from the English longitudinal study of ageing. J. Am. Geriatr. Soc. 66, 1823–1829. doi: 10.1111/jgs.15456, 30098017

[ref24] Dawson H. N. Ferreira A. Eyster M. V. Ghoshal N. Binder L. I. Vitek M. P. (2001). Inhibition of neuronal maturation in primary hippocampal neurons from tau deficient mice. J. Cell Sci. 114, 1179–1187. doi: 10.1242/jcs.114.6.1179, 11228161

[ref25] de Gomez Barreda E. Perez M. Gomez Ramos P. de Cristobal J. Martin-Maestro P. Moran A. . (2010). Tau-knockout mice show reduced GSK3-induced hippocampal degeneration and learning deficits. Neurobiol. Dis. 37, 622–629. doi: 10.1016/j.nbd.2009.11.017, 20004245

[ref26] Diez I. Ortiz-Teran L. Ng T. S. C. Albers M. W. Marshall G. Orwig W. . (2024). Tau propagation in the brain olfactory circuits is associated with smell perception changes in aging. Nat. Commun. 15:4809. doi: 10.1038/s41467-024-48462-3, 38844444 PMC11156945

[ref27] Donovan L. J. Brewer C. L. Bond S. F. Laslavic A. M. Pena Lopez A. Colman L. . (2025). Aging and injury drive neuronal senescence in the dorsal root ganglia. Nat. Neurosci. 28, 985–997. doi: 10.1038/s41593-025-01954-x, 40369367 PMC12081305

[ref28] Ehrlich J. R. Goldstein J. Swenor B. K. Whitson H. Langa K. M. Veliz P. (2022). Addition of vision impairment to a life-course model of potentially modifiable dementia risk factors in the US. JAMA Neurol. 79, 623–626. doi: 10.1001/jamaneurol.2022.0723, 35467745 PMC9039828

[ref29] Fischer I. (2022). Evolutionary perspective of big tau structure: 4a exon variants of MAPT. Front. Mol. Neurosci. 15:1019999. doi: 10.3389/fnmol.2022.1019999, 36533137 PMC9755724

[ref30] Fischer I. Baas P. W. (2020). Resurrecting the mysteries of big tau. Trends Neurosci. 43, 493–504. doi: 10.1016/j.tins.2020.04.007, 32434664 PMC7999525

[ref31] Fischer I. Connors T. Bouyer J. Jin Y. (2024). The unique properties of big tau in the visual system. Cytoskeleton (Hoboken) 81, 488–499. doi: 10.1002/cm.21875, 38761116

[ref32] Fletcher P. D. Downey L. E. Golden H. L. Clark C. N. Slattery C. F. Paterson R. W. . (2015). Pain and temperature processing in dementia: a clinical and neuroanatomical analysis. Brain 138, 3360–3372. doi: 10.1093/brain/awv276, 26463677 PMC4620514

[ref33] Galton F. (1883). Inquiries into human faculty and its development: Dent. London: Macmillan and co.

[ref34] Gilbert A. N. Yamazaki K. Beauchamp G. K. Thomas L. (1986). Olfactory discrimination of mouse strains (Mus musculus) and major histocompatibility types by humans (Homo sapiens). J. Comp. Psychol. 100, 262–265. 3769446

[ref35] Gleeson M. Timmins F. (2004). The use of touch to enhance nursing care of older person in long-term mental health care facilities. J. Psychiatr. Ment. Health Nurs. 11, 541–545. doi: 10.1111/j.1365-2850.2004.00757.x 15450020

[ref36] Growdon M. E. Schultz A. P. Dagley A. S. Amariglio R. E. Hedden T. Rentz D. M. . (2015). Odor identification and Alzheimer disease biomarkers in clinically normal elderly. Neurology 84, 2153–2160. doi: 10.1212/WNL.0000000000001614, 25934852 PMC4451046

[ref37] Hampel H. Lista S. Mango D. Nistico R. Perry G. Avila J. . (2019). Lithium as a treatment for Alzheimer's disease: the systems pharmacology perspective. J. Alzheimer's Dis 69, 615–629. doi: 10.3233/JAD-190197, 31156173

[ref38] Harada A. Oguchi K. Okabe S. Kuno J. Terada S. Ohshima T. . (1994). Altered microtubule organization in small-calibre axons of mice lacking tau protein. Nature 369, 488–491. doi: 10.1038/369488a0, 8202139

[ref39] Hardy C. J. Marshall C. R. Golden H. L. Clark C. N. Mummery C. J. Griffiths T. D. . (2016). Hearing and dementia. J. Neurol. 263, 2339–2354. doi: 10.1007/s00415-016-8208-y, 27372450 PMC5065893

[ref40] Hart N. J. Koronyo Y. Black K. L. Koronyo-Hamaoui M. (2016). Ocular indicators of Alzheimer's: exploring disease in the retina. Acta Neuropathol. 132, 767–787. doi: 10.1007/s00401-016-1613-6, 27645291 PMC5106496

[ref41] Hedger N. Naselaris T. Kay K. Knapen T. (2026). Vicarious body maps bridge vision and touch in the human brain. Nature 650, 173–181. doi: 10.1038/s41586-025-09796-0, 41299177 PMC12872459

[ref42] Helgason A. Yngvadottir B. Hrafnkelsson B. Gulcher J. Stefansson K. (2005). An Icelandic example of the impact of population structure on association studies. Nat. Genet. 37, 90–95. doi: 10.1038/ng1492, 15608637

[ref43] Ho W. L. Leung Y. Tsang A. W. So K. F. Chiu K. Chang R. C. (2012). Review: tauopathy in the retina and optic nerve: does it shadow pathological changes in the brain? Mol. Vis. 18, 2700–2710. 23170062 PMC3501278

[ref44] Iqbal K. Liu F. Gong C. X. (2016). Tau and neurodegenerative disease: the story so far. Nat. Rev. Neurol. 12, 15–27. doi: 10.1038/nrneurol.2015.225, 26635213

[ref45] Ittner L. M. Ke Y. D. Delerue F. Bi M. Gladbach A. van Eersel J. . (2010). Dendritic function of tau mediates amyloid-beta toxicity in Alzheimer's disease mouse models. Cell 142, 387–397. doi: 10.1016/j.cell.2010.06.036, 20655099

[ref46] Jackson F. (1982). Epiphenomenal Qualia. Philos. Q. 32, 127–136.

[ref47] Jessen F. Amariglio R. E. Buckley R. F. van der Flier W. M. Han Y. Molinuevo J. L. . (2020). The characterisation of subjective cognitive decline. Lancet Neurol. 19, 271–278. doi: 10.1016/S1474-4422(19)30368-0, 31958406 PMC7062546

[ref48] Jones M. W. Hunt T. (2023). Electromagnetic-field theories of qualia: can they improve upon standard neuroscience? Front. Psychol. 14:1015967. doi: 10.3389/fpsyg.2023.1015967, 37325753 PMC10267331

[ref49] Just D. K. C. (2017). Was Kandinsky a Synaesthete? Examining his writings and other evidence. Multisens. Res. 30, 447–460. doi: 10.1163/22134808-00002547, 31287076

[ref50] Kehl M. S. Mackay S. Ohla K. Schneider M. Borger V. Surges R. . (2024). Single-neuron representations of odours in the human brain. Nature 634, 626–634. doi: 10.1038/s41586-024-08016-5, 39385026 PMC11485236

[ref51] Kim H. J. Kim B. H. Kim D. K. Kim H. Choi S. H. Kim D. H. . (2024a). Phosphorylated tau in the taste buds of Alzheimer's disease mouse models. Exp Neurobiol 33, 202–214. doi: 10.5607/en24004, 39266476 PMC11411091

[ref52] Kim H. J. Tadros B. Liang Y. H. Kim Y. Lasagna-Reeves C. Sonn J. Y. . (2024b). TYK2 regulates tau levels, phosphorylation and aggregation in a tauopathy mouse model. Nat. Neurosci. 27, 2417–2429. doi: 10.1038/s41593-024-01777-2, 39528671 PMC11614740

[ref53] Kimura T. Whitcomb D. J. Jo J. Regan P. Piers T. Heo S. . (2014). Microtubule-associated protein tau is essential for long-term depression in the hippocampus. Philos. Trans. R. Soc. Lond. Ser. B Biol. Sci. 369:20130144. doi: 10.1098/rstb.2013.0144, 24298146 PMC3843876

[ref54] Klein J. Yan X. Johnson A. Tomljanovic Z. Zou J. Polly K. . (2021). Olfactory impairment is related to tau pathology and Neuroinflammation in Alzheimer's disease. J. Alzheimer's Dis 80, 1051–1065. doi: 10.3233/JAD-201149, 33646153 PMC8044007

[ref55] Kolo F. B. Lu S. Beiser A. S. Francis L. Melo van Lent D. Gireud-Goss M. . (2025). Hearing loss, brain structure, cognition, and dementia risk in the Framingham heart study. JAMA Netw. Open 8:e2539209. doi: 10.1001/jamanetworkopen.2025.39209, 41191359 PMC12590305

[ref56] Koronyo Y. Rentsendorj A. Mirzaei N. Regis G. C. Sheyn J. Shi H. . (2023). Retinal pathological features and proteome signatures of Alzheimer's disease. Acta Neuropathol. 145, 409–438. doi: 10.1007/s00401-023-02548-2, 36773106 PMC10020290

[ref57] Lee V. M. Goedert M. Trojanowski J. Q. (2001). Neurodegenerative tauopathies. Annu. Rev. Neurosci. 24, 1121–1159. doi: 10.1146/annurev.neuro.24.1.1121, 11520930

[ref58] Lee A. T. C. Richards M. Chan W. C. Chiu H. F. K. Lee R. S. Y. Lam L. C. W. (2020). Higher dementia incidence in older adults with poor visual acuity. J. Gerontol. A Biol. Sci. Med. Sci. 75, 2162–2168. doi: 10.1093/gerona/glaa036, 32043518 PMC7566398

[ref59] Livingston G. Huntley J. Liu K. Y. Costafreda S. G. Selbaek G. Alladi S. . (2024). Dementia prevention, intervention, and care: 2024 report of the lancet standing commission. Lancet 404:39096926, 572–628. doi: 10.1016/S0140-6736(24)01296-0 39096926

[ref60] Lorenzi R. M. Palesi F. Castellazzi G. Vitali P. Anzalone N. Bernini S. . (2020). Unsuspected involvement of spinal cord in Alzheimer disease. Front. Cell. Neurosci. 14:6. doi: 10.3389/fncel.2020.00006, 32082122 PMC7002560

[ref61] Macknin J. B. Higuchi M. Lee V. M. Trojanowski J. Q. Doty R. L. (2004). Olfactory dysfunction occurs in transgenic mice overexpressing human tau protein. Brain Res. 1000, 174–178. doi: 10.1016/j.brainres.2004.01.047, 15053964

[ref62] Masters C. L. Bateman R. Blennow K. Rowe C. C. Sperling R. A. Cummings J. L. (2015). Alzheimer's disease. Nat. Rev. Dis. Primers 1:15056. doi: 10.1038/nrdp.2015.56 27188934

[ref63] Meftah S. Cavallini A. Murray T. K. Jankowski L. Bose S. Ashby M. C. . (2024). Synaptic alterations associated with disrupted sensory encoding in a mouse model of tauopathy. Brain Commun 6:fcae134. doi: 10.1093/braincomms/fcae134, 38712321 PMC11073755

[ref64] Mellone M. Kestoras D. Andrews M. R. Dassie E. Crowther R. A. Stokin G. B. . (2013). Tau pathology is present in vivo and develops in vitro in sensory neurons from human P301S tau transgenic mice: a system for screening drugs against tauopathies. J. Neurosci. 33, 18175–18189. doi: 10.1523/JNEUROSCI.4933-12.2013, 24227726 PMC3828468

[ref65] Meyer A. Leigh D. Bagg C. E. (1954). A rare presenile dementia associated with cortical blindness (Heidenhain's syndrome). J. Neurol. Neurosurg. Psychiatry 17, 129–133. doi: 10.1136/jnnp.17.2.129, 13163704 PMC503171

[ref66] Moltrasio J. Rubinstein W. (2025). The soundtrack of memory: the effect of music on emotional memory in Alzheimer's disease and older adults. Memory 33, 1266–1280. doi: 10.1080/09658211.2025.2573267, 41105815

[ref67] Moriguchi Y. Watanabe R. Sakata C. Zeleznikow-Johnston A. Wang J. Saji N. . (2025). Comparing color qualia structures through a similarity task in young children versus adults. Proc. Natl. Acad. Sci. USA 122:e2415346122. doi: 10.1073/pnas.2415346122, 40067901 PMC11929397

[ref68] Nadhimi Y. Llano D. A. (2021). Does hearing loss lead to dementia? A review of the literature. Hear. Res. 402:108038. doi: 10.1016/j.heares.2020.108038, 32814645 PMC9336511

[ref69] Nicholas A. P. (2013). Dual immunofluorescence study of citrullinated proteins in Alzheimer diseased frontal cortex. Neurosci. Lett. 545, 107–111. doi: 10.1016/j.neulet.2013.04.028, 23648390 PMC3731154

[ref70] Nothias F. Boyne L. Murray M. Tessler A. Fischer I. (1995). The expression and distribution of tau proteins and messenger RNA in rat dorsal root ganglion neurons during development and regeneration. Neuroscience 66, 707–719. 7644032 10.1016/0306-4522(94)00598-y

[ref71] Oblinger M. M. Argasinski A. Wong J. Kosik K. S. (1991). Tau gene expression in rat sensory neurons during development and regeneration. J. Neurosci. 11, 2453–2459. 1714493 10.1523/JNEUROSCI.11-08-02453.1991 PMC6575521

[ref72] O'Riordan K. J. Hu N. W. Rowan M. J. (2018). Ass facilitates LTD at Schaffer collateral synapses preferentially in the left Hippocampus. Cell Rep. 22, 2053–2065. doi: 10.1016/j.celrep.2018.01.085, 29466733

[ref73] Orpwood R. (2017). Information and the origin of qualia. Front. Syst. Neurosci. 11:22. doi: 10.3389/fnsys.2017.00022, 28484376 PMC5399078

[ref74] Paciello F. Rinaudo M. Longo V. Cocco S. Conforto G. Pisani A. . (2021). Auditory sensory deprivation induced by noise exposure exacerbates cognitive decline in a mouse model of Alzheimer's disease. eLife 10:e70908. doi: 10.7554/eLife.70908, 34699347 PMC8547960

[ref75] Pallas-Bazarra N. Jurado-Arjona J. Navarrete M. Esteban J. A. Hernandez F. Avila J. . (2016). Novel function of tau in regulating the effects of external stimuli on adult hippocampal neurogenesis. EMBO J. 35:27198172, 1417–1436. doi: 10.15252/embj.201593518 PMC4876034 27198172

[ref76] Panza F. Solfrizzi V. Logroscino G. (2015). Age-related hearing impairment-a risk factor and frailty marker for dementia and AD. Nat. Rev. Neurol. 11, 166–175. doi: 10.1038/nrneurol.2015.12, 25686757

[ref77] Park S. Y. Kim M. J. Kim H. L. Kim D. K. Yeo S. W. Park S. N. (2018). Cognitive decline and increased hippocampal p-tau expression in mice with hearing loss. Behav. Brain Res. 342, 19–26. doi: 10.1016/j.bbr.2018.01.003, 29317248

[ref78] Pennanen L. Welzl H. D'Adamo P. Nitsch R. M. Gotz J. (2004). Accelerated extinction of conditioned taste aversion in P301L tau transgenic mice. Neurobiol. Dis. 15, 500–509. doi: 10.1016/j.nbd.2003.11.020, 15056457

[ref79] Ramachandran V. S. Hirstein W. (1997). Three laws of qualia: what neurology tells us about the biological functions of consciousness. J. Conscious. Stud. 4, 429–457.

[ref80] Regan P. Piers T. Yi J. H. Kim D. H. Huh S. Park S. J. . (2015). Tau phosphorylation at serine 396 residue is required for hippocampal LTD. J. Neurosci. 35, 4804–4812. doi: 10.1523/JNEUROSCI.2842-14.2015, 25810511 PMC4389589

[ref81] Risacher S. L. Tallman E. F. West J. D. Yoder K. K. Hutchins G. D. Fletcher J. W. . (2017). Olfactory identification in subjective cognitive decline and mild cognitive impairment: association with tau but not amyloid positron emission tomography. Alzheimers Dement (Amst) 9, 57–66. doi: 10.1016/j.dadm.2017.09.001, 29159268 PMC5675709

[ref82] Rodriguez L. Joly S. Zine-Eddine F. Mdzomba J. B. Pernet V. (2020). Tau modulates visual plasticity in adult and old mice. Neurobiol. Aging 95, 214–224. doi: 10.1016/j.neurobiolaging.2020.07.024, 32858248

[ref83] Schmidt M. L. Zhukareva V. Perl D. P. Sheridan S. K. Schuck T. Lee V. M. . (2001). Spinal cord neurofibrillary pathology in Alzheimer disease and Guam parkinsonism-dementia complex. J. Neuropathol. Exp. Neurol. 60, 1075–1086. doi: 10.1093/jnen/60.11.1075, 11706937

[ref84] Shi H. Mirzaei N. Koronyo Y. Davis M. R. Robinson E. Braun G. M. . (2024). Identification of retinal oligomeric, citrullinated, and other tau isoforms in early and advanced AD and relations to disease status. Acta Neuropathol. 148:3. doi: 10.1007/s00401-024-02760-8, 38980423 PMC11233395

[ref85] Skokowski P. (2022). Sensing qualia. Front. Syst. Neurosci. 16:795405. doi: 10.3389/fnsys.2022.795405, 35359622 PMC8962373

[ref86] Spence C. (2015). Just how much of what we taste derives from the sense of smell? Flavour 4:30. doi: 10.1186/s13411-015-0040-2

[ref87] Steinbach S. Hundt W. Vaitl A. Heinrich P. Forster S. Burger K. . (2010). Taste in mild cognitive impairment and Alzheimer's disease. J. Neurol. 257, 238–246. doi: 10.1007/s00415-009-5300-6, 19727902

[ref88] Swords G. M. Nguyen L. T. Mudar R. A. Llano D. A. (2018). Auditory system dysfunction in Alzheimer disease and its prodromal states: a review. Ageing Res. Rev. 44, 49–59. doi: 10.1016/j.arr.2018.04.001, 29630950 PMC9664450

[ref89] Thomson R. S. Auduong P. Miller A. T. Gurgel R. K. (2017). Hearing loss as a risk factor for dementia: a systematic review. Laryngoscope Investig Otolaryngol 2, 69–79. doi: 10.1002/lio2.65, 28894825 PMC5527366

[ref90] Tian Q. Bilgel M. Moghekar A. R. Ferrucci L. Resnick S. M. (2022). Olfaction, cognitive impairment, and PET biomarkers in community-dwelling older adults. J. Alzheimer's Dis 86, 1275–1285. doi: 10.3233/JAD-210636, 35180111 PMC12163179

[ref91] Tsuchiya N. Saigo H. Phillips S. (2022). An adjunction hypothesis between qualia and reports. Front. Psychol. 13:1053977. doi: 10.3389/fpsyg.2022.1053977, 37077507 PMC10107370

[ref92] Tuerde D. Kimura T. Miyasaka T. Furusawa K. Shimozawa A. Hasegawa M. . (2018). Isoform-independent and -dependent phosphorylation of microtubule-associated protein tau in mouse brain during postnatal development. J. Biol. Chem. 293, 1781–1793. doi: 10.1074/jbc.M117.798918, 29196605 PMC5798307

[ref93] Tye M. (2006). Absent qualia and the mind-body problem. Philos. Rev. 115, 139–168. doi: 10.1215/00318108-115-2-139

[ref94] Valles-Saiz L. Domene-Serrano I. Picher A. J. Perez M. Garcia-Escudero V. Hernandez F. . (2025). Structure and function of full-length tau. PLoS One 20:e0335251. doi: 10.1371/journal.pone.0335251, 41171737 PMC12578172

[ref95] Walkiewicz G. Ronisz A. Van Ginderdeuren R. Lemmens S. Bouwman F. H. Hoozemans J. J. M. . (2024). Primary retinal tauopathy: a tauopathy with a distinct molecular pattern. Alzheimers Dement. 20, 330–340. doi: 10.1002/alz.13424, 37615275 PMC10916964

[ref96] Wang S. E. Wu C. H. (2021). Tau phosphorylation and cochlear apoptosis cause hearing loss in 3xTg-AD mouse model of Alzheimer's disease. Chin. J. Phys. 64, 61–71. doi: 10.4103/CJP.CJP_79_20, 33938816

[ref97] Wang H. W. Wysocki C. J. Gold G. H. (1993). Induction of olfactory receptor sensitivity in mice. Science 260, 998–1000. 8493539 10.1126/science.8493539

[ref98] Wang H. F. Zhang W. Rolls E. T. Alzheimer's Disease Neuroimaging, I Li Y. Wang L. . (2022). Hearing impairment is associated with cognitive decline, brain atrophy and tau pathology. EBioMedicine 86:104336. doi: 10.1016/j.ebiom.2022.104336, 36356475 PMC9649369

[ref99] Wood R. M. Garcia Z. Daniels N. Landon S. M. Humayun S. Lee H. G. . (2020). Selective peripheral taste dysfunction in APP/PS1 mutant transgenic mice. J. Alzheimer's Dis 76, 613–621. doi: 10.3233/JAD-200376, 32538852 PMC7643563

[ref100] Xu W. Zhang C. Li J. Q. Tan C. C. Cao X. P. Tan L. . (2019). Age-related hearing loss accelerates cerebrospinal fluid tau levels and brain atrophy: a longitudinal study. Aging (Albany NY) 11, 3156–3169. doi: 10.18632/aging.101971, 31118310 PMC6555452

[ref101] Zaretskaya N. (2024). When sensory input meets spontaneous brain activity. Trends Neurosci. 47, 749–750. doi: 10.1016/j.tins.2024.08.010 39218722

[ref102] Zhang Y. Yang Y. Hu Z. Zhu M. Qin S. Yu P. . (2022). Long-term depression-inducing low frequency stimulation enhances p-Tau181 and p-Tau217 in an age-dependent manner in live rats. J. Alzheimer's Dis 89, 335–350. doi: 10.3233/JAD-220351, 35871344 PMC9484260

[ref103] Zheng M. Yan J. Hao W. Ren Y. Zhou M. Wang Y. . (2022). Worsening hearing was associated with higher β-amyloid and tau burden in age-related hearing loss. Sci. Rep. 12:10493. doi: 10.1038/s41598-022-14466-6, 35729211 PMC9212197

